# Significant Stenosis Without Thrombus: Is It the Third Most Common Morphology of Acute Coronary Syndrome?

**DOI:** 10.1016/j.cjco.2025.01.019

**Published:** 2025-01-30

**Authors:** Hiroyoshi Mori, Myong Hwa Yamamoto, Takuya Mizukami, Nobuaki Kobayashi, Kohei Wakabayashi, Seita Kondo, Teruo Sekimoto, Takehiko Sambe, Sakiko Yasuhara, Tomoyo Sugiyama, Tsunekazu Kakuta, Satoru Mitomo, Sunao Nakamura, Masamichi Takano, Taishi Yonetsu, Tomotaka Dohi, Jun Yamashita, Junichi Yamaguchi, Shigeki Kimura, Takumi Higuma, Makoto Natsumeda, Yuji Ikari, Satoru Suwa, Hiroshi Suzuki, Toshiro Shinke, TACTICS investigators

**Affiliations:** a Division of Cardiology, Department of Internal Medicine, Showa University Fujigaoka Hospital, Kanagawa, Japan; b Clinical Research Institute for Clinical Pharmacology & Therapeutics, Showa University, Tokyo, Japan; c Division of Clinical Pharmacology, Department of Pharmacology, Showa University School of Medicine, Tokyo, Japan; d Department of Cardiology, Nippon Medical School Chiba Hokusoh Hospital, Chiba, Japan; e Division of Cardiology, Cardiovascular Center, Showa University Koto-Toyosu Hospital, Tokyo, Japan; f Division of Cardiology, Department of Internal Medicine, Showa University School of Medicine, Tokyo, Japan; g Division of Cardiovascular Medicine, Tsuchiura Kyodo General Hospital, Ibaraki, Japan; h Department of Cardiovascular Medicine, New Tokyo Hospital, Chiba, Japan; i Department of Cardiovascular Medicine, Tokyo Medical and Dental University, Tokyo, Japan; j Department of Cardiovascular Biology and Medicine, Juntendo University Graduate School of Medicine, Tokyo, Japan; k Department of Cardiology, Tokyo Medical University Hospital, Tokyo, Japan; l Department of Cardiology, Tokyo Women's Medical University, Tokyo, Japan; m Department of Cardiology, Yokohama Minami Kyosai Hospital, Kanagawa, Japan; n Division of Cardiology, Department of Internal Medicine, Kawasaki Municipal Tama Hospital, Kanagawa, Japan; o Department of Cardiology, Tokai University School of Medicine, Kanagawa, Japan; p Department of Cardiovascular Medicine, Juntendo University Shizuoka Hospital, Shizuoka, Japan

## Abstract

**Background:**

Significant stenosis without thrombus (SSWT) is sometimes observed in patients with acute coronary syndrome (ACS). However, its incidence and clinical features remains unclear.

**Method:**

This substudy of the TACTICS registry included patients with ACS (n = 702) undergoing emergency percutaneous coronary intervention using optical coherence tomography. Using this registry data, we compared the clinical features of SSWT in patients with ACS. Major adverse cardiac events were defined as cardiac death, nonfatal myocardial infarction, heart failure, or ischemia-driven revascularization at 1 year.

**Results:**

Plaque rupture (PR; n = 411, 59.1%) and plaque erosion (PE; n = 178, 25.6%) were the 2 major morphologies, followed by SSWT (n = 64, 9.1%) and calcified nodule (CN; n = 28, 4.0%). Patients with SSWT were slightly older than those with PR and PE, but younger than those with CN. In the SSWT, non-ST elevation myocardial infarction was the main type of ACS, followed by unstable angina and ST-elevation myocardial infarction (63%, 22%, and 15%, respectively). Lesions were less complex with a lower proportion of type B2 or C, shorter procedure time, lower proportion of multivessel disease, and lower syntax score, which led to a lower incidence of major adverse cardiac events.

**Conclusion:**

SSWT was the third most common morphology of ACS, with clinical features different from those of PR, PE, and CN.

## Résumé

**Contexte:**

Une sténose significative sans thrombus (SSST) est parfois observée chez les patients souffrant d'un syndrome coronarien aigu (SCA). Cependant, son incidence et ses caractéristiques cliniques restent floues.

**Méthode:**

Cette sous-étude du registre TACTICS a porté sur des patients atteints de SCA (n = 702) ayant subi une intervention coronarienne percutanée d'urgence par tomographie en cohérence optique. En utilisant les données de ce registre, nous avons comparé les caractéristiques cliniques d'une SSST chez les patients atteints de SCA. Les événements cardiaques indésirables majeurs (ECIM) ont été définis comme une mort cardiaque, un infarctus du myocarde non fatal, une insuffisance cardiaque ou une revascularisation entraînée par une ischémie à 1 an.

**Résultats:**

La rupture de plaque (RP ; n = 411, 59,1 %) et l'érosion de plaque (EP ; n = 178, 25,6 %) étaient les deux principales morphologies répertoriées, suivies par la SSST (n = 64, 9,1 %) et la présence d'un nodule calcifié (NC ; n = 28, 4,0 %). Les patients atteints d'une SSST étaient légèrement plus âgés que ceux avec RP et EP, mais plus jeunes que ceux présentant un NC. Chez les patients atteints d'une SSST, l'infarctus du myocarde sans élévation du segment ST était le principal type de SCA, suivi de l'angine instable et de l'infarctus du myocarde avec élévation du segment ST (63 %, 22 % et 15 %, respectivement). Les lésions étaient moins complexes, avec une proportion plus faible de type B2 ou C, une durée d'intervention plus courte, une proportion plus faible de maladie multivaisseaux et un score SYNTAX (complexité et sévérité des lésions) plus faible, ce qui a conduit à une incidence plus faible d'ECIM.

**Conclusion:**

Le SSST était la troisième morphologie la plus fréquente en cas de SCA, avec des caractéristiques cliniques différentes de celles de la RP, de l'EP et du NC.

Acute coronary syndrome (ACS) is a life-threating disease worldwide.^1^ Based on the lesion morphology of ACS, its mechanism is generally explained by plaque rupture (PR), plaque erosion (PE), and calcified nodule (CN).^2^, ^3^, ^4^, ^5^, ^6^, ^7^ PR is the most common coronary thrombosis caused by disruption of thin-cap fibroatheroma (TCFA).^6^^,^^7^ PE is the second most common cause of coronary thrombosis and is characterized by thrombosis in the absence of intimal disruption.^2^^,^^4^^,^^5^ CN is a relatively rare cause of coronary thrombosis occurring in older age with features of a lesion with fibrous cap disruption and lumina thrombus associated with eruptive, dense, calcific nodules.^4^, ^5^, ^6^^,^^8^ Although these 3 morphologies are major causes of coronary thrombosis, it is not well known that significant stenosis without thrombus (SSWT) is sometimes observed in sudden coronary deaths.^6^ Thus, the majority of studies focusing on the mechanism of ACS using optical coherence tomography (OCT) have rarely described SSWT.^3^^,^^4^

In this study, we aimed to explore the clinical features and prognosis of SSWT using the TACTICS registry, which is a prospective multicenter registry that collects data from ACS patients who underwent emergency percutaneous coronary intervention (PCI) under OCT guidance.^5^

## Methods

This was a substudy of the TACTICS registry (Tokyo, Kanagawa, Chiba, Shizuoka, and Ibaraki active OCT applications for ACS), which was an investigator-initiated, prospective, multicenter, observational study conducted at 22 hospitals in Japan between November 2019 and April 2021. The rationale and design of the TACTICS registry have been previously published, and the study was registered at UMIN-CTR (UMIN000039050).^9^ Briefly, patients with ACS within 24 hours of the onset of symptoms who underwent emergency PCI under OCT guidance were included.^5^ ACS included ST-segment elevation myocardial infarction (STEMI), non–ST-segment elevation myocardial infarction (NSTEMI), and unstable angina (UA). The study protocol was approved by the institutional ethics committee of each hospital and was registered in the University Hospital Medical Information Network Clinical Trials Registry in Japan (UMIN-CTR, ID 000039050). This study was performed in accordance with the principles of the Declaration of Helsinki and written informed consent was obtained from all patients.

Emergency PCI under OCT guidance was performed using the standard techniques. The culprit lesion was determined by each PCI operator based on a combination of electrocardiographic changes, coronary angiography, and luminal thrombus recognized by OCT. Thrombus aspiration and/or gentle predilation with a ≤2.0-mm balloon was accepted to obtain prompt recanalization and optimal preprocedural OCT image acquisition if needed. Coronary angiograms were interpreted offline at an independent core laboratory (Cardio Score Japan; Tokyo, Japan). The SYNTAX score was used to assess lesion complexity, and pre- and postprocedural coronary flow were assessed using the TIMI flow grade.

The ILUMIEN OPTIS system (Abbott Vascular Inc, Santa Clara, CA) and Dragonfly OPTIS or Dragonfly OpStar imaging catheter (Abbott Vascular Inc) were used to acquire the OCT images. Contrast media or low-molecular-weight dextran were used as flushing agents during the OCT image acquisition. Offline software was used to analyze the OCT images at an independent OCT core laboratory (Kobe Cardiovascular Core Analysis Laboratory, Hyogo, Japan). Qualitative analysis of the baseline OCT images was performed by 2 independent experienced interventional cardiologists who were blinded to all clinical data, except for angiography and the procedure, and the plaque morphology of the culprit lesion was assessed. Consensus reading was obtained via discussion at a conference attended by at least 6 experienced analysts in case of disagreement.

The plaque morphology was defined as follows: PR was defined as coronary thrombosis with disruption of the fibrous cap overlying a lipid plaque; PE was defined as a coronary thrombosis with an intact fibrous cap; CN was defined as the eruption of calcific nodules into the lumen with disruption of the fibrous cap; and SSWT was defined based on the presence of ACS without coronary thrombosis but with significant stenosis (>75% luminal narrowing). Other morphologic features included spontaneous coronary artery dissection, coronary ectasia/aneurysm, vasospasm, and coronary embolism.

The present study focused on the SSWT in comparison with PR, PE, and CN. Major adverse cardiac events (MACE) included cardiovascular death, myocardial infarction, heart failure, and ischemia-driven revascularization. Heart failure was defined when treatment was initiated or intensified, specifically for heart failure. Clinical characteristics and MACE were compared.

Continuous variables were expressed as median values (25th-75th percentile), whereas categorical variables were expressed as percentages. Variables with non-normal distributions were compared using the Wilcoxon test. Categorical variables were analyzed using the χ^2^ test, as appropriate. A Kaplan-Meier survival analysis was used to evaluate the cumulative incidence of the clinical outcomes. JMP 17 (SAS Institute, Cary, NC) was used to perform the statistical analyses. Statistical significance was set at *P* <0.05. The interobserver kappa coefficients for PR, PE, and CN was 0.890.

## Results

**Figure 1:**
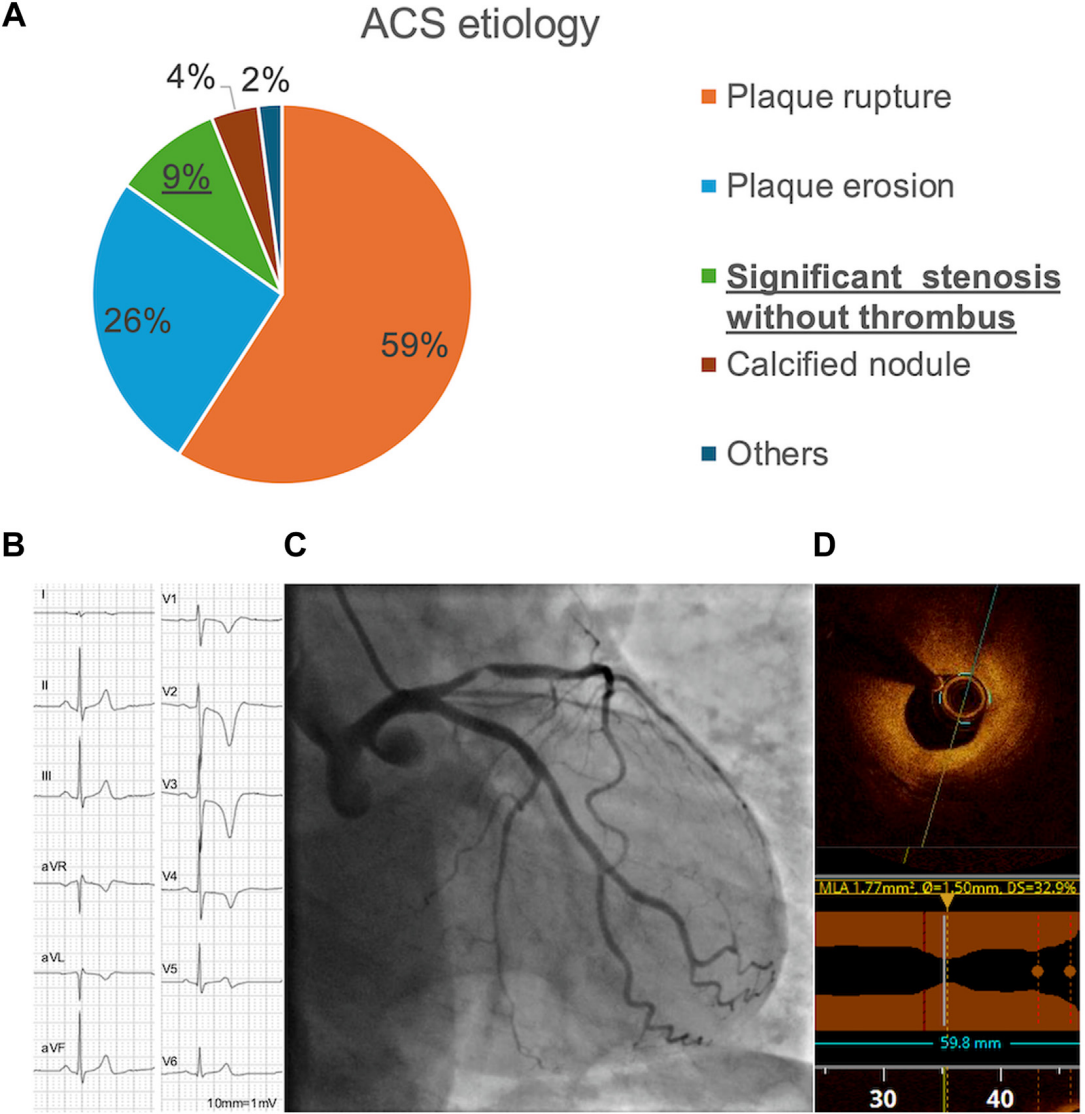
(**A**) Distribution of plaque morphologies in the culprit lesions of ACS. A representative case of significant stenosis in a 75-year-old man is shown in panels **B to D**. (**B** and **C**) The patient visited our hospital because of lung cancer but ST changes in his electrocardiogram and a positive troponin test led us to perform emergent intervention. (**D**) OCT shows significant stenosis without thrombus. ACS, acute coronary syndrome; OCT, ocular coherence tomography.

After excluding those with inadequate images (n = 7), 695 patients were enrolled in the main study. PR was the most frequent morphology (n = 411; 59.1%), followed by PE (n = 178; 25.6%), SSWT (n = 64; 9.1%), and CN (n = 28; 4.0%) (Figure 1). The remaining patients (n = 15) had coronary spasm (n = 7; 1.0%), ectasia (n = 3; 0.4%), embolism (n = 3; 0.4%), and spontaneous coronary dissection (n = 1; 0.1%). Table 1 shows the patient characteristics. The median age of the SSWT group was 70 years, which was much lower than that of the CN group but slightly higher than those of the PR and PE groups. Accordingly, the proportion of male patients was 73% in the SSWT group, which was lower in comparison to the PR (81%) and PE (84%) groups but higher in comparison to the CN group (64%). The prevalence of diabetes was the highest in the CN group (50%), followed by the PR (33%), SSWT (30%), and PE (23%) groups. Active smoking was most frequently observed in the PE group (43%), followed by the PR (35%), SSWT (19%), and CN (14%) groups. The proportion of patients undergoing hemodialysis was very high in the CN group (21%) but not in the other groups. Low-density lipoprotein cholesterol levels were higher in PR and PE, followed by SSWT and CN. History of malignancy did not differ between the groups. With the exception of antidiabetic drugs and β-blocker, most of the medications at discharge did not differ between the groups.

**Table 1:** Patient characteristics

	Significant stenosis without thrombus (n = 64)	Plaque rupture (n = 411)	Plaque erosion (n = 178)	Calcified nodule (n = 28)	*P* value
Age, y	70 (59-75)	68 (57-76)	65 (52-73)	80 (72-82)	<0.01
Male	47 (73)	332 (81)	150 (84)	18 (64)	0.04
Hypertension	39 (61)	274 (67)	112 (63)	20 (71)	0.62
Diabetes	19 (30)	135 (33)	41 (23)	14 (50)	0.01
Dyslipidemia	43 (67)	235 (57)	104 (58)	16 (57)	0.51
Active smoking	12 (19)	143 (35)	77 (43)	4 (14)	<0.01
Hemodialysis	0 (0)	6 (1)	4 (2)	6 (21)	<0.01
Atrial fibrillation	5 (8)	15 (4)	6 (3)	1 (4)	0.43
Previous MI	3 (5)	17 (4)	5 (3)	2 (7)	0.69
LDL cholesterol, mg/dL	117 (91-133)	127 (104-154)	128 (107-153)	103 (79-120)	<0.01
HDL cholesterol, mg/dL	48 (38-60)	48 (40-57)	47 (41-54)	49 (42-58)	0.73
TG, mg/dL	129 (82-211)	110 (73-175)	120 (69-192)	96 (61-131)	0.19
HbA1c, %	6.0 (5.6-6.7)	6.0 (5.7-6.7)	6.0 (5.7-6.5)	6.4 (5.5-7.3)	0.65
History of malignancy	2 (3)	27 (7)	4 (2)	1 (4)	0.13
Statin	64 (100)	405 (99)	174 (98)	26 (97)	0.51
Ezetimibe	10 (16)	57 (14)	34 (19)	2 (7)	0.26
Antidiabetic drug	17 (27)	119 (29)	33 (19)	12 (44)	<0.01
SGLT2i	8 (13)	73 (18)	23 (13)	4 (15)	0.42
Calcium blocker	16 (25)	54 (13)	30 (17)	5 (19)	0.09
ACEi/ARB	48 (75)	312 (76)	131 (74)	15 (56)	0.13
β-Blocker	32 (50)	291 (71)	129 (72)	18 (67)	0.01
Diuretics	3 (5)	30 (7)	8 (4)	1 (4)	0.51
OAC	6 (9)	33 (8)	12 (7)	2 (7)	0.16
Antiplatelet	64 (100)	410 (100)	176 (99)	26 (96)	0.08

ACEi, angiotensin converting enzyme inhibitor; ARB, angiotensin II receptor blocker; HbA1c, glycated hemoglobin; HDL, high-density lipoprotein; LDL, low-density lipoprotein; OAC, oral anti-coagulation; SGLT2i, Sodium-glucose transporter 2 inhibitor; TG, triglycerides.

Continuous variables are expressed as median (25th-75th percentile), and categorical variables are expressed as n (%).

Table 2 shows the characteristics of ACS. The type of ACS in the SSWT group was mostly NSTEMI or UAP whereas two-thirds of ACS was STEMI in the PR, PE, and CN groups. Thus, the peak CK and BNP levels were lowest in the SSTW group, and EF was the best in the SSWT group. The procedure time in the CN group was very long relative to the SSWT, PR, and PE groups. The target vessel was mostly the LAD, which was similar to that in the PE group but dissimilar to those in the PR and CN groups. As expected, the initial TIMI flow grade was mostly 2 or 3 in the SSWT group. The SSWT group showed the lowest proportions of type B2 or C. Killip class III or IV, need of IABP, planning for staged PCI, and the syntax score was the lowest in SSWT. The SSWT group had the lowest rates of requirement for thrombus aspiration or predilation.

**Table 2:** Characteristics of acute coronary syndrome

	Significant stenosis without thrombus (n = 64)	Plaque rupture (n = 411)	Plaque erosion (n = 178)	Calcified nodule (n = 28)	*P* value
Type of ACS					<0.01
STEMI	10 (15)	299 (73)	106 (60)	16 (57)	
NSTEMI	41 (63)	103 (25)	65 (37)	10 (36)	
UAP	14 (22)	9 (2)	7 (4)	2 (7)	
SBP, mm Hg	137 (123-159)	138 (121-156)	142 (125-164)	129 (115-155)	0.04
DBP, mm Hg	82 (71-93)	86 (71-98)	88 (75-104)	75 (61-90)	<0.01
HR	78 (65-90)	77 (65-90)	81 (68-92)	70 (60-86)	0.11
Hemoglobin, g/dL	14.2 (13.1-15.1)	14.5 (13.2-15.8)	14.8 (13.6-15.9)	13.3 (11.0-14.3)	<0.01
Peak CK, U/L	159 (90-263)	512 (145-1991)	319 (142-1121)	266 (82-625)	<0.01
BNP, pg/mL	36 (22-69)	43 (16-127)	43 (20-121)	167 (49-518)	<0.01
EF, %	62 (57-66)	57 (48-63)	57 (49-62)	59 (51-62)	<0.01
Procedure time, min	65 (43-85)	70 (47-95)	62 (40-93)	110 (82-134)	<0.01
Contrast volume, mL	156 (122-217)	158 (125-228)	162 (130-220)	170 (119-265)	0.65
Target vessel					<0.01
LM artery	1 (2)	2 (1)	1 (1)	1 (4)	
LAD artery	41 (64)	198 (48)	112 (63)	12 (43)	
LCX artery	6 (9)	38 (9)	20 (11)	0 (0)	
RC artery	16 (25)	173 (42)	45 (25)	15 (54)	
TIMI flow grade (initial)					<0.01
0	2 (3)	187 (46)	70 (39)	9 (32)	
1	8 (13)	64 (16)	21 (12)	3 (11)	
2	23 (36)	94 (23)	42 (24)	6 (21)	
3	31 (48)	66 (16)	45 (25)	10 (36)	
Type B2 or C	33 (52)	270 (66)	104 (58)	23 (82)	<0.01
Killip class III or IV	0 (0)	30 (7.3)	5 (2.8)	6 (21)	<0.01
IABP	1 (2)	28 (7)	5 (3)	4 (14)	0.02
Plan for staged PCI	8 (13)	106 (26)	41 (23)	10 (36)	0.06
Syntax score	8 (6-12)	12 (8-20)	12 (7-18)	15 (11-26)	<0.01
Aspiration	3 (5)	206 (50)	65 (36)	6 (21)	<0.01
Predilation	7 (11)	128 (31)	45 (25)	15 (54)	<0.01
Postdilation	44 (69)	271 (66)	113 (63)	19 (68)	0.87
Stentless	4 (6)	3 (1)	4 (2)	5 (18)	<0.01

ACS, acute coronary syndrome; BNP, B-type natriuretic peptide; CK, creatine kinase; DBP, diastolic blood pressure; EF, ejection fraction; HR, heart rate; IABP, intra-aortic balloon pumping; LAD, left anterior descending; LCX, left circumflex; LM, left main; NSTEMI, non–ST-elevation myocardial infarction; PCI, percutaneous coronary intervention; RC, right coronary; SBP, systolic blood pressure; STEMI, ST-elevation myocardial infarction; UAP, unstable angina pectoralis.

Continuous variables are expressed as median (25th-75th percentile), and categorical variables are expressed as n (%).

**Figure 2:**
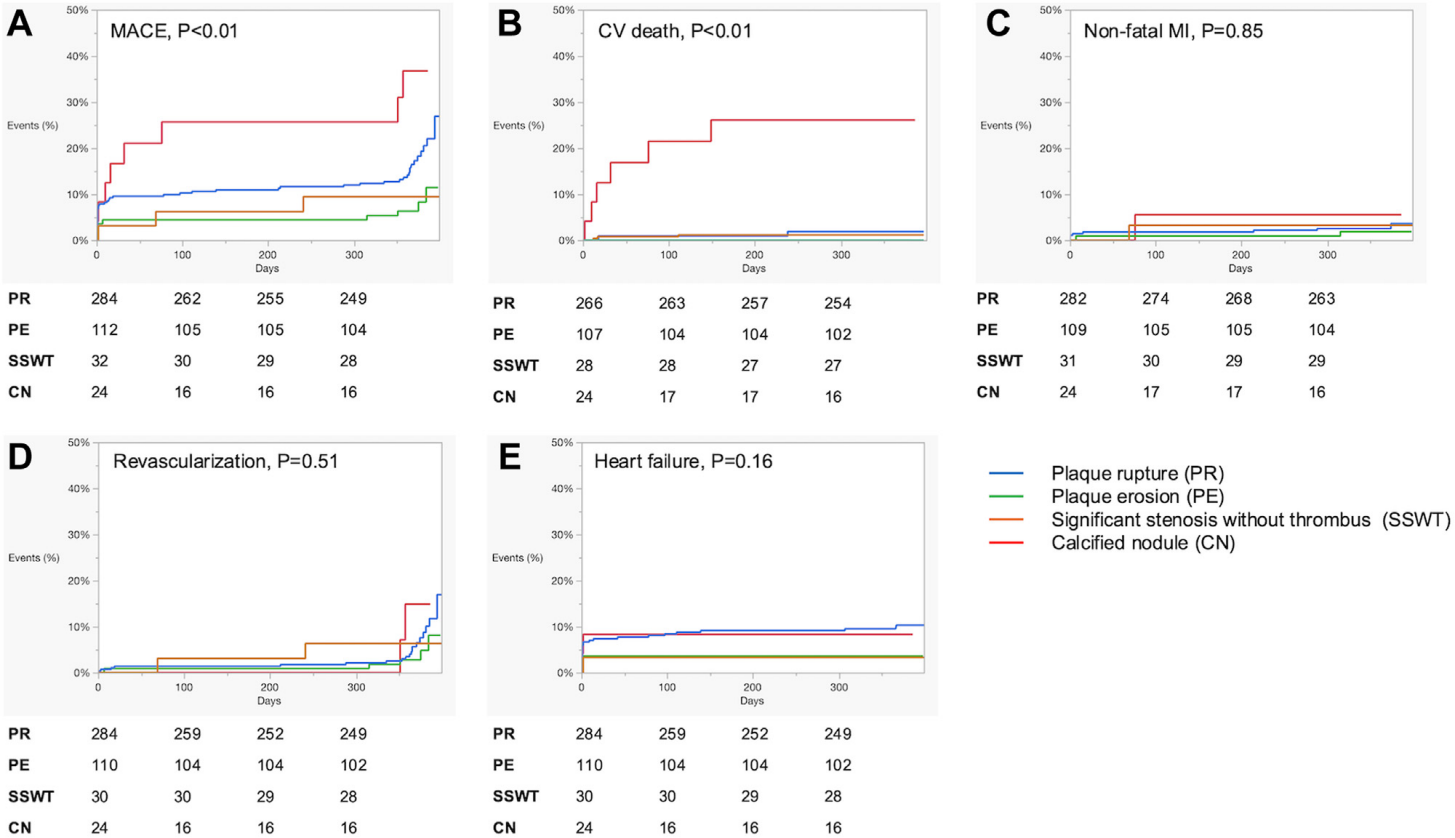
Kaplan-Meier survival curve showing the cumulative incidence of (**A**) MACE, (**B**) cardiac death, (**C**) nonfatal MI, (**D**) ischemia-driven revascularization, and (**E**) heart failure. MACE, major adverse cardiac events; MI, myocardial infarction.

Table 3 shows the characteristics of OCT. The PR group had the highest rate of lipidic plaque (lipid max arc >180°), the highest macrophage grade, and the highest rate of cholesterol crystals, whereas these values were moderate in the PE and SSWT (44%) groups and were lowest in the CN group. Calcified plaque (calcium max arc >180°) was a distinct feature of CN (100%), which was much less frequent in SSWT, PR, and PE. Layered plaque were similarly observed among SSWT, PR, and PE but it was much less in CN. There were no significant differences in postinterventional minimum stent area, reference area, expansion rate, and microchannel. The cumulative incidence of MACE is expressed in Figure 2, showing that CN was the worst followed by PR. PE and SSWT showed similar MACE at 1 year. Cardiovascular death was distinctly high in CN, whereas most of the events were minimum in PE and SSWT.

**Table 3:** OCT characteristics

	Significant stenosis without thrombus (n = 64)	Plaque rupture (n = 411)	Plaque erosion (n = 178)	Calcified nodule (n = 28)	*P* value
Lipid max arc >180°	28 (44)	376 (91)	93 (52)	5 (18)	<0.01
Calcification max arc >180°	11 (17)	35 (9)	18 (10)	28 (100)	<0.01
Postintervention MSA, mm^2^	6.0 (4.6-7.2)	5.9 (4.4-7.6)	5.8 (4.4-7.3)	5.0 (4.7-6.1)	0.20
Proxreference area, mm	8.7 (6.9-13.1)	5.3 (6.4-10.7)	7.9 (6.1-10.2)	6.8 (5.2-7.8)	0.12
Dist reference area, mm	5.4 (4.0-7.8)	5.5 (4.0-7.8)	5.6 (4.3-8.0)	4.7 (3.4-6.3)	0.54
Expansion rate, %	74 (62-84)	74 (64-87)	75 (63-86)	72 (63-91)	0.99
Macrophage grade					<0.01
0	12 (19)	10 (2)	21 (12)	9 (32)	
1	11 (17)	16 (4)	19 (11)	2 (7)	
2	24 (38)	104 (25)	54 (30)	7 (25)	
3	15 (23)	208 (51)	68 (38)	7 (25)	
4	2 (3)	72 (18)	16 (9)	3 (10)	
Cholesterol crystal	28 (44)	251 (61)	88 (49)	9 (32)	<0.01
Microchannel	25 (39)	194 (47)	94 (53)	8 (29)	0.10
Layered	49 (77)	284 (69)	144 (81)	10 (36)	<0.01

Dist, distal; MSA, minimum stent area; OCT, optical coherence tomography; Prox, proximal.

Continuous variables are expressed as median (25th-75th percentile), and categorical variables are expressed as n (%).

## Discussion

The SSWT was the third most common morphology of ACS in the present study. Patients with SSWT were slightly older than those with PR and PE, with a relatively lower prevalence of active smoking. Clinical presentations were mostly NSTEMI or UAP in patients with SSWT. In terms of plaque characteristics, the lipid component was similar to that of PE, but the degree of calcification was slightly greater than that observed in PR and PE. The cumulative incidence of MACE was lower in the SSWT and PE groups than in the CN and PR groups.

The pathophysiology of SSWT remains to be elucidated and requires further discussion. We speculate that SSWT is not a single event because significant stenosis itself is more commonly observed in chronic coronary syndrome (CCS).^10^ Coronary spasm, systemic condition increasing myocardial oxygen consumption (type II MI), and rapid plaque progression are considerable contributors to SSWT. Coronary spasm in addition to significant stenosis can be a trigger of ACS.^11^ Coronary spasm is often reported in Japanese patients.^12^ Indeed, it has been reported that coronary spasm was provoked in 72.5% of Japanese patients with AMI who underwent acetylcholine provocation test within a few weeks after onset of AMI.^13^ Therefore, we believe that coronary spasm plays an important role in SSWT. Alternatively, ACS can occur when myocardial oxygen consumption increases, which is known as type II MI.^14^ Rapid plaque progression can trigger ACS. Asymptomatic subclinical acute coronary syndrome can also cause significant subclinical ACS. It is known that asymptomatic PR or PE can occur and that these conditions are frequently observed in both ACS and CCS.^15^^,^^16^ These lesions often form layered plaques, leading to plaque progression. Indeed, in our study, the layered plaques in the SSWT group were comparable to those in the PR and PE groups. Alternatively, intraplaque hemorrhage, another mechanism of rapid plaque progression, may sometimes occur.^17^ Another possibility is microvascular dysfunction in addition to significant stenosis.^18^ Accordingly, ACS may occur when significant stenosis is combined with coronary spasm or an increase in myocardial oxygen consumption, rapid plaque progression, microvascular dysfunction, or when these situations are mixed together.^19^ It seems that patients with SSWT are very heterogenous, but we believe that this is one aspect of ACS.

Studies focusing on the etiology of ACS using OCT are limited. Some studies have focused on STEMI, and have mainly reported on PR, PE, and CN.^4^ Other studies have described the etiology of ACS as just PR or an intact fibrous cap without going deeper into the intact fibrous cap.^3^^,^^20^^,^^21^ We speculate some might be diagnosed with PE when the lesion has significant stenosis even in the absence of a thrombus. If we maintain that mindset, we cannot know why and how ACS occurs in the lesions without thrombi. It is likely that SSWT was underrecognized at the beginning of the OCT studies. A study focusing on PE reported that tight stenosis was observed in 19 of 405 lesions.^22^ The pathophysiology of these patients may be similar to SSWT in our study. An autopsy study showed that SSWT accounted for a quarter of cases of sudden coronary death.^6^ Therefore, we believe that SSWT can be one of the plaque morphologies in ACS, although its pathophysiology remains unclear.

The PCI procedure for the SSWT was relatively simple. As shown in our data, PCI procedure time and contrast volume were minimal in SSWT and PE groups. As these lesions had no thrombus, the need for thrombus aspiration and the need for predilation were both minimal in SSWT. Thus, most SSWT lesions were treated with direct stenting after OCT imaging. Stentless strategies using drug-coated balloons without stent implantation are becoming an option these days.^22^ In our data, the proportion of patients treated with the stentless strategy was numerically greater in SSWT than in PR and PE, although the overall total number of patients treated with the stentless strategy was small overall.

Guideline-directed medical treatments is likely suitable for SSWT.^23^ The cumulative incidence of MACE was minimal in the SSWT and PE groups. We believe that a substantial proportion of SSWT involved healed rupture or healed erosion, as described above. Thus, lipid-lowering therapy remains a key therapy for the secondary prevention of SSWT, as we all know.^23^ We also speculated that coronary spasms may be involved in some SSWT. Attempting a provoked spasm test and/or the use of calcium channel blockers should be considered if coronary spasm is suspected.^11^

The present study had several limitations. Selection bias may have existed in the study population because the TACTICS registry was an observational study, and the indication for OCT guidance was left to the operators. Indeed, OCT guidance was performed in 702 of the 1702 patients in this study.^5^ Thrombus aspiration and/or gentle predilation using a small balloon was allowed before the initial OCT, which may have altered the lesion morphology. Although the findings were judged by multiple observers, the OCT-based assessment of underlying causes of ACS was not supported by the histological definition of these mechanisms. Although we speculated that coronary spasm plays an important role in SSWT, acetylcholine provocation test was not performed unless the primary physician determines that it was necessary.

## Conclusion

SSWT is one of the major lesion morphologies in ACS. SSWT occurred less frequently than PR and PE but more frequently than CN in our registry. The PCI procedure for SSWT was relatively less demanding, and the clinical outcome of SSWT was also relatively better. Recognizing SSWT as a culprit lesion morphology in ACS may widen our understanding of ACS.
